# Establishing scientific confidence: human biological relevance of reconstructed human respiratory epithelium (RHRE) for assessing respiratory effects

**DOI:** 10.3389/ftox.2026.1847761

**Published:** 2026-06-10

**Authors:** Nuria Roldan, Emily N. Reinke, Helena T. Hogberg, Andreas O. Stucki, Monique M. Perron, Amy J. Clippinger

**Affiliations:** 1 PETA Science Consortium International e.V., Stuttgart, Germany; 2 General Dynamics Information Technology (GDIT), Reston, VA, United States; 3 National Toxicology Program Interagency Center for the Evaluation of Alternative Toxicological Methods (NICEATM), National Institute of Environmental Health Sciences, Durham, NC, United States; 4 US Environmental Protection Agency, Office of Pesticide Programs, Washington, DC, United States

**Keywords:** human relevance, *in vitro*, inhalation toxicity testing, MucilAir, new approach methodologies (NAMs), reconstructed human respiratory epithelium (RHRE), respiratory toxicity, lung tissues

## Abstract

*In vitro* test methods can be used to evaluate the effects of inhaled substances on the human respiratory tract. Scientists are increasingly using these tools due to interest in maximizing reliability and human-relevance, and therefore, the ability to protect human health. Among these *in vitro* models, reconstructed human respiratory epithelium (RHRE) is designed to mimic aspects of the biology of the human respiratory tract and to reflect mechanisms perturbed by different insults, including chemical exposure. In this paper, the human biological relevance of RHRE, in particular the MucilAir model, is assessed to gauge confidence in the use of RHRE-based test methods to evaluate respiratory effects. While the paper focuses on MucilAir, many of the key concepts also apply to other RHRE models. Key features of MucilAir, such as the presence of relevant primary human cells that produce mucus and have beating cilia, demonstrate its relevance to human biology. The model is compatible with a range of assays (such as those assessing cytotoxicity, cell viability, and cellular barrier integrity) that measure key events that may occur in humans following exposure. In this paper, we also present case studies of how these models have been used to predict the toxicity of inhaled substances. Overall, RHRE can be used to provide a quantitative, mechanism-based understanding of the potential effects of chemicals on the human respiratory tract.

## 1 Introduction

Inhalation is the major route through which humans are exposed to substances present in the air, including gases and aerosols (e.g., liquids, particles, or fibers). To protect human health, regulatory agencies worldwide have requirements to establish whether the unintentional or intentional inhalation of chemicals, pesticides, drugs, or other substances may cause detrimental effects. These effects may include portal-of-entry effects in the respiratory tract as well as systemic toxicity (Clippinger et al., 2018a).

To meet regulatory requirements, inhalation toxicity tests have often been conducted using rats. However, differences in the anatomy and physiology of the human and rat respiratory tracts limit the precision with which rats can reliably predict human effects (Stucki et al., 2024). As a result, *in silico*, *in chemico*, *in vitro*, and *ex vivo* approaches have been developed (e.g. (Clippinger et al., 2018b; Mercier et al., 2019; Shrestha et al., 2020; Ladics et al., 2021; McGee Hargrove et al., 2021; Patel et al., 2021; 2023; Zamprogno et al., 2021; Sørli et al., 2022; Boisleve et al., 2025; de Ávila et al., 2025)). Cell-based *in vitro* models have been designed to mimic aspects of the biology of the human respiratory tract and mechanisms of inhalation toxicity in humans. Most of these *in vitro* methods make use of human cells, thereby overcoming the inherent species differences between rats and humans. Human cell–based *in vitro* models have the potential to reliably and rapidly characterize the effects of inhaled substances. They can also provide a better mechanistic understanding of how an inhaled substance exerts its toxic effects in humans.

*In vitro* reconstructed human respiratory epithelium (RHRE) is a specialized organotypic cell culture type that has features of the *in situ* human respiratory epithelium. RHRE models are designed to reflect a specific region of the human respiratory tract (e.g., nasal, tracheal, or bronchial) and comprise relevant cell types with different functions. RHRE models can be generated using immortalized, primary, or induced pluripotent stem cell (iPSC)-derived cells. RHRE can also be used as part of microphysiological systems (MPS), either by directly seeding cells within a system (Huh et al., 2010; Stucki et al., 2015; Zamprogno et al., 2021; Park et al., 2024), or by connecting commercially available RHRE as part of single or multiorgan MPS (Bovard et al., 2018; Schimek et al., 2020; Phan et al., 2023). Commercially available RHRE models are available around the world, can be procured from various manufacturers (Table 1), and are being used to develop methods for different purposes.

**TABLE 1:** Commercially available *in vitro* reconstructed human respiratory epithelium (RHRE) (alphabetized by provider). This table includes RHRE comprised of immortalized, primary, or induced pluripotent stem cells-derived respiratory cells but does not include microphysiological systems.

Name	Provider^a^	Region of the respiratory tract modelled	Cell types
^AX^iAECs	AlveoliX (Switzerland)	Alveolar	Immortalized human alveolar epithelial cells
SoluAirway	Biosolution (South Korea)	Tracheobronchial	Primary human bronchial epithelial cells
AlveolAir	Epithelix (Switzerland)	Alveolar	Primary human alveolar epithelial cells (type I and II) and endothelial cells
MucilAir	Epithelix	Nasal, tracheal, or bronchial	Primary human nasal, tracheal, or bronchial epithelial cells
MucilAir^−^HF	Epithelix	Nasal, tracheal, or bronchial	Primary human nasal, tracheal, or bronchial epithelial cells, and fibroblasts
SmallAir	Epithelix	Small airway (bronchiolar)	Primary human small airway epithelial cells
SmallAir^−^HF	Epithelix	Small airway (bronchiolar)	Primary human small airway epithelial cells and fibroblasts
HiTrach	HiLung (Japan)	Tracheal	Human iPSC-derived tracheal epithelial cells
HiAlv	HiLung	Alveolar	Human iPSC-derived alveolar epithelial cells
ImmuLUNG	ImmuONE (United Kingdom)	Alveolar	Immortalized human alveolar epithelial cells and alveolar macrophage-like cells
ALIsens	Invitrolize (Luxembourg)	Alveolar	Immortalized human alveolar epithelial cells line (A549), immortalized human endothelial cells (EA.hy926), immortalized human macrophage-like cells (THP-1 differentiated with PMA), and immortalized human dendritic-like cells (THP-1)
EpiNasal	MatTek (United States of America)	Nasal	Primary human nasal epithelial cells
EpiAirway	MatTek	Tracheobronchial	Primary human tracheal or bronchial epithelial cells
EpiAirwayFT	MatTek	Tracheobronchial	Primary human tracheal or bronchial epithelial cells and stromal fibroblasts
EpiAlveolar	MatTek	Alveolar	Primary human alveolar epithelial cells (type I and II), pulmonary fibroblasts, and pulmonary endothelial cells. Option to add macrophage cell line (THP-1)
Human lung small airway epithelial cell model	Newcells biotech (United Kingdom)	Small airway (bronchiolar)	Primary human small airway epithelial cells
Nasal epithelial well-differentiated cultures	University of North Carolina (UNC) – Airway BioCore (United States of America)	Nasal	Primary human nasal epithelial cells
Large airway epithelial (LAE) well-differentiated cultures	UNC – Airway BioCore	Tracheobronchial	Primary human tracheal or bronchial epithelial cells
Small airway epithelial (SAE) well-differentiated cultures	UNC – Airway BioCore	Small airway (bronchiolar)	Primary human small airway epithelial cells

^a^ Websites of the providers listed (accessed 11 Feb 2026).

AlveoliX: https://alveolix.com/

Biosolution: http://keraskin.co.kr/

Epithelix: https://epithelix.com/

HiLung: https://hilung.com/

ImmuOne: https://immuone.com/

Invitrolize: https://invitrolize.com/

MatTek: https://mattek.com/

Newcells Biotech: https://newcellsbiotech.co.uk/

University of North Carolina–Airway BioCore: https://www.med.unc.edu/airwaybiocore/

Scientific confidence in a method can be evaluated using principles outlined in well-established international frameworks (van der Zalm et al., 2022; ICCVAM, 2024). Key elements of these frameworks include biological relevance, fitness-for-purpose (or context of use), technical characterization, independent review, and data transparency and integrity. For the purposes of this review, we evaluated the human biological relevance of RHRE to help understand their suitability for assessing potential chemical effects in humans. The use of these scientific confidence-building frameworks allows for a consistent, transparent, and science-based means of evaluation. This paper reviews the human biological relevance of RHRE, building the scientific confidence needed for the adoption of modern scientific tools that best protect human health. This paper includes two regulatory case studies of the application of RHRE-based methods to assess respiratory effects. However, evaluating specific methods is outside the scope of this review and would require detailed consideration of the method, protocol, and context of use.

In particular, we focused on the RHRE, MucilAir, as a prototypic example because it has been available for almost two decades (Caulfuty and Huang, 2009), has been extensively characterized, has been used to produce a wealth of toxicological data, and is the only RHRE model thus far used to generate data used in regulatory applications. However, many of the concepts discussed here may apply to other RHRE, particularly, those of the conducting airway. These include RHRE with tissue structure and cell composition similar to MucilAir (e.g., EpiAirway and SoluAirway; Table 1). Work is ongoing to build scientific confidence in these models and support their regulatory use (Jackson et al., 2018; Hwang et al., 2021; Lee et al., 2021; Wallace et al., 2023; Choe et al., 2026). Due to the different anatomical location they represent, their cell composition, and tissue structure, RHRE of the small airway and pulmonary regions (Table 1) are not specifically discussed in this review.

## 2 Human biological relevance of the RHRE, MucilAir

The biological relevance of a method is determined by demonstrating alignment between the biology of the species of interest (in this case humans) and the model system, as well as the ability to capture relevant mechanisms of toxicity. MucilAir is made from primary human cells (see summary of the evaluation of MucilAir’s human biological relevance in Table 2). It is available from different anatomical sites, namely, the nasal, tracheal, and bronchial regions, and the brand name, MucilAir, is used interchangeably for all three models. MucilAir can be generated using cells from a single donor (there are more than 30 individual nasal, 10 tracheal, and more than 35 bronchial donors available) or a pool of 5 (bronchial) or 14 (nasal) donors, offering versatility. Single donor RHRE offer mechanistic information about donor-specific and non-specific responses. They also allow for studying response levels for different demographic groups (e.g., by sex or age) or genetic backgrounds, and avoid allogenic variability of mixed donors. MucilAir single donor is also available using cells from healthy individuals or those with certain pathologies (i.e., chronic obstructive pulmonary disease, asthma, cystic fibrosis, or allergic rhinitis) and smoking histories. On the other hand, MucilAir-pool provides an averaged donor response, minimizing the impact of donor-specific variability across studies and experimental repetitions. MucilAir-pool can be sourced in larger quantities than MucilAir single donor, making it well-suited for assessments requiring higher throughput or many repetitions.

**TABLE 2:** Summary of the human biological relevance evaluation of the RHRE, MucilAir.

Descriptor	MucilAir description	Significance
Cell type	Human	Representative of the species of interest. Contains basal, ciliated, and mucus-producing goblet cells. Fibroblasts can be added on request
Region of the respiratory tract	Nasal, tracheal, or bronchial	Allows for selection of the region that is most likely to be targeted by and/or most sensitive to the test substance
Donors	Tissues can be made from individual or pooled donors, and from healthy donors or those with pathologies	Offers versatility and possibility to stratify populations of interest
Culture condition	Air-liquid interface	Mimics situation of the human respiratory tract
Stability	After differentiation, stable for several months to 1 year	Allows for evaluation of chronic exposure and repeated dosing
Culture media	Chemically defined	Eliminates variability due to animal-derived ingredients and uses human specific growth factors and hormones
Structure	Pseudostratified, and includes a similar proportion of relevant cells and tissue thickness as compared to humans	Allows for direct comparison of histopathology images to human *in vivo* tissues
Functionality	Captures key features of the human respiratory tract, including barrier function through intercellular tight junction formation, mucus production and mucus viscoelasticity, beating cilia, active ion transport, liquid absorption, metabolic activity, and the release of cytokines, chemokines, and metalloproteinases. Possesses key receptors for viral/bacterial infection	Allows for a quantitative evaluation of key markers of toxicity and disease modeling

MucilAir is differentiated and grown at the air-liquid interface (ALI), which mimics the organization of the human respiratory tract that is exposed to air on one side and receives nutrients from the other (Lacroix et al., 2018). After 4–6 weeks of differentiation, MucilAir remains functional for several months (Baxter et al., 2015; Hoffmann et al., 2018). MucilAir is cultured in chemically defined media containing human-derived growth factors and hormones, eliminating the variability associated with the use of fetal bovine serum and other animal-derived supplements, which vary from batch to batch (Stival et al., 2025).

In humans and in MucilAir, nasal, tracheal, and bronchial epithelia are made of the same three types of cells (basal, ciliated, and mucus-producing goblet cells). MucilAir contains these three cell types in a similar proportion to that found in humans (Huang et al., 2011). When stromal-epithelial interactions or fibrosis are of interest, MucilAir is also available in a version that contains primary stromal fibroblasts (MucilAir-HF) (Kim et al., 2022; Moreau et al., 2022; Hervé et al., 2024). MucilAir models representing different regions of the respiratory tract are available. This allows for the selection of the model that is most likely to be targeted by the test substance (e.g., as determined by computational fluid dynamic modeling) and/or the most sensitive region. For example, substances primarily affecting the lower respiratory tract (e.g., those causing emphysema or pulmonary edema) may be more relevant to assess in a model of the lower respiratory tract, as opposed to using a tracheobronchial RHRE (Sauer et al., 2013). However, comparative studies of nasal and tracheobronchial RHRE suggest that testing on each region may produce equivalent results in their response to xenobiotics (Iskandar et al., 2013; Moreau et al., 2022) and functional assays, such as transepithelial permeability (Hoffmann et al., 2018). This is not surprising, as human nasal epithelial cells and nasal RHRE have been proposed as a surrogate for bronchial epithelium. This is due to their similar characteristics and composition of secreted mucus (Pohl et al., 2025), and the easier accessibility (e.g., from nasal brushings (McDougall et al., 2008; Iskandar et al., 2013)). Nevertheless, differences in the sensitivity of these models to specific xenobiotics have not been systematically characterized. Differences among MucilAir types may be relevant for specific chemical classes, particularly chemicals subject to local metabolic (de)toxification (Baxter et al., 2015; Kelty et al., 2022), or in their response to different biological processes (e.g., viral infections (Pizzorno et al., 2020)). Due to the structural and functional similarities among MucilAir types, for the purpose of this review, MucilAir is used interchangeably to refer to nasal, tracheal, or bronchial.

**FIGURE 1:**
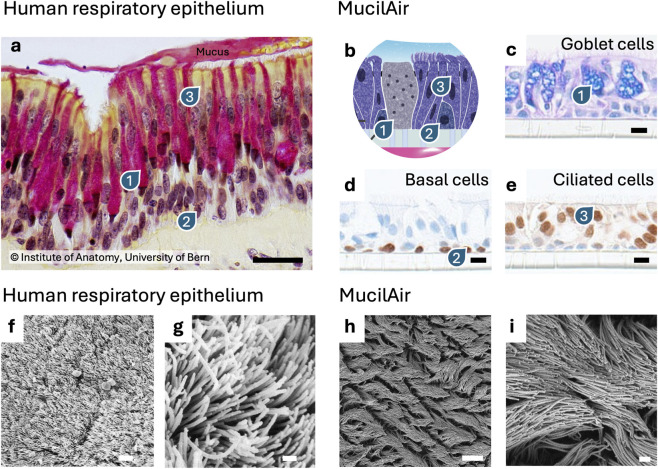
Histology of human respiratory tissue and MucilAir. **(a)** Cross-section micrograph of human nasal epithelium stained with hematoxylin (nuclei), mucicarmine (mucus), and trichrome (connective tissue) for human bronchial epithelium illustrating mucus-producing goblet cells (1), basal cells, (2) and ciliated cells (3). Scale bar is 25 μm. **(b)** Schematic tissue structure of MucilAir. **(c)** Histological cross-sections of MucilAir with hematoxylin-eosin and alcian blue (mucus) staining, **(d)** immunostaining of basal cells (P63), and **(e)** ciliated cells (FoxJ1). Scale bar is 10 μm. Scanning electron micrographs of cilia obtained from human respiratory epithelium **(f,g)**, and MucilAir **(h,i)**. Scale bar for **(f,h)** is 10 μm; scale bar for **(g,i)** is 1 μm. **(a)** is reproduced from MedSurf, Institute of Medical Education, University of Bern, with permission. **(f,g)** are reproduced with permission from the publisher. **(b–e)** and **(h,i)** are kindly provided by Epithelix.

Histologically, MucilAir shows a pseudostratified structure comparable to that of human nasal, tracheal, and bronchial respiratory epithelia (Figure 1) and is able to replicate clinical histological findings suggestive of airway tissue remodeling due to respiratory irritation, including goblet cell hyperplasia (Czekala et al., 2021; Chapman et al., 2023) and squamous metaplasia (Paudel et al., 2023; Recio et al., 2025). Goblet cells present in MucilAir, produce and secrete airway surface liquid (ASL), consisting of a fluid periciliary layer and a mucus layer (Figure 1). The thickness of its mucus (10 µm) and epithelial cell layer (30–40 µm) resembles that of the human respiratory tract (Tarran et al., 2001; Song et al., 2009). MucilAir-produced ASL and purified mucus have been compared to human sputum to assess their biological relevance. Human sputum and MucilAir purified mucus share similar mucin contents, as demonstrated by rheological properties. However, MucilAir mucus has a more fluid-like character, requiring less stress to flow, compared to human sputum. This more fluid character is attributed to a lower degree of complexity and crosslinking caused by differences in its composition (Esteban Enjuto et al., 2024). Proteomic analysis showed that MucilAir ASL shares nearly 50% of the same proteins with human sputum, at varying concentrations (Baxter et al., 2015). Human sputum contains molecules from diverse sources, including immune cells and secretions from various regions of the respiratory tract. However, MucilAir is exclusively derived from the respiratory epithelium, which explains the different composition. To our knowledge, no quantitative comparisons between human, RHRE, and rat mucus compositions have been conducted. Generally, it is known that rat mucus has significant contributions from secretory serous cells, which are less frequent in humans and secrete a product with lower viscosity than human mucus. Additionally, rat mucus is deficient in proteins which are abundant in human mucus (e.g., MUC5AC) (Stucki et al., 2024). Mucus remains an area of research interest, and human-derived RHRE mucus presents an opportunity to better understand factors affecting secretion and composition, providing mechanistic information relevant to toxicity assessments.

MucilAir has beating cilia (Behrsing et al., 2022), an essential function of human airway epithelial cells that enables mucociliary clearance (MCC). In humans, MCC is responsible for the transport of mucus in a proximal direction (towards the nose and mouth), where it is either swallowed or excreted by coughing or sneezing. Beating cilia are present in RHRE, enabling the study of ciliary function and clearance rates *in vitro*. However, the MCC process cannot be fully replicated *in vitro* due to the physical limitations imposed by the cell culture support. To address this limitation, RHRE-based test methods involve washing steps to clear excess mucus and applied test substances that cannot be absorbed. Motile cilia with a directed beating pattern are present on at least 60% of the total MucilAir surface area (Lodes et al., 2020). In humans, there is limited evidence on the total ciliation area in the respiratory tract. Roth et al. estimated 30%–50% ciliation area in the human trachea based on previous studies (Mercer et al., 1994; Okuda et al., 2021), but reported up to 86% in human tracheobronchial explants based on a more recent study (Roth et al., 2025). The reported typical cilia beat frequency of MucilAir (Behrsing et al., 2022) is slightly higher than that reported for human tracheobronchial explants (Roth et al., 2025); however, RHRE ciliation, cilia properties, and clearance rates have been shown to vary depending on the temperature and cell culture media used. In one study, the RHRE evaluated (with one exception) exhibited similar CBF, but lower clearance rates, compared to human explants (MucilAir was not included in this study) (Roth et al., 2025). At the time of this publication, cilia properties and clearance rates in MucilAir have not been directly compared with human *in vivo or ex vivo* samples in a single study.

MucilAir has been demonstrated to be metabolically competent in defined contexts. For example, the exposure of these tissues to certain xenobiotics will cause an activation of metabolic enzymes similar to what can be observed in humans (Iskandar et al., 2013; Baxter et al., 2015; Oesch et al., 2019; Bovard et al., 2020). However, an in-depth, comprehensive study of its full metabolic competency has not yet been published. Metabolic enzyme and activity levels may vary depending on the substrate and region of the respiratory tract modelled. For example, MucilAir has shown inducibility and activity of phase I cytochrome P450 (CYP) enzymes upon exposure to different substances (e.g., dioxin (2, 3, 7, 8 tetrachlorodibenzo-p-dioxin; TCDD), tobacco products (Iskandar et al., 2013; Baxter et al., 2015), and coumarin (Baxter et al., 2015)). However, a study addressing the biotransformation of naphthalene in MucilAir revealed some limitations in phase II metabolism (Kelty et al., 2022). The reason for this may be attributable to site specific metabolism (i.e., increased sensitivity of Club cells, not present in MucilAir), different donor susceptibilities, loss of metabolic competency over culturing time, and/or the short exposure protocol (1-h) used in the study (Kelty et al., 2022). Thus, future studies addressing the metabolic competency of RHRE require careful consideration of donor susceptibilities, enzyme functionality, and testing protocols. Importantly, RHRE made from human cells avoid interspecies differences in metabolism that might occur in the absence of cells from the species of interest.

As in human respiratory epithelium, MucilAir has barrier function characterized by tight junction formation and transepithelial electrical resistance (TEER) values above 200 Ω.cm^2^ (Mercier et al., 2019). It displays active ion transport, preserving the native activity of key ionic channels—such as cystic fibrosis transmembrane conductance regulator (CFTR), epithelial sodium channel (ENaC), and sodium/potassium (Na/K) ATPase (Huang et al., 2011)—enabling the regulation of water absorption at the air-liquid interface (Baiocco et al., 2021). MucilAir also expresses active ATP-binding cassette (ABC) transporters (Mercier et al., 2019). MucilAir demonstrated molecule efflux for rhodamine and chlorothiazide, mediated by P-glycoprotein (P-gp) and breast cancer resistance protein (BCRP), respectively, which was inhibited by specific inhibitors (Mercier et al., 2019). The assay used in the study to demonstrate drug transport was useful for detecting a subset of transporters and substrates; however, it showed limitations in detecting multidrug resistance-associated protein (MRP)-1-mediated efflux. This was attributed to a technical limitation of the assay and substrate used (which was susceptible to being transported by different MRPs isoforms) and not a definitive outcome regarding the capabilities of MucilAir for this receptor (Mercier et al., 2019). The receptor typically shows functionality in other pulmonary *in vitro* models, including RHRE (Rotoli et al., 2020; Simon et al., 2025), further indicating a technical limitation of the assay/substrate. Another study showed that MucilAir can be used to study molecule absorption and can differentiate between molecules with high and low respiratory absorption, which are transported through passive (e.g., antipyrine, caffeine, naproxen, and propranolol) or paracellular routes (e.g., atenolol, mannitol, and PEG-400), respectively (Reus et al., 2014). Altogether, these data show that MucilAir expresses active transporters and forms a tight but selectively permeable barrier. Thus, it can serve as a model to study absorption, which provides useful information that can be fed into physiologically based kinetic (PBK) models to study systemic toxicity. MucilAir releases relevant cytokines, chemokines, and metalloproteinases in response to stimuli in a regulated manner (Huang et al., 2011).

Therefore, MucilAir can be used to evaluate key markers of toxicity, including barrier integrity (e.g., TEER measurement), cytotoxicity (e.g., LDH release), metabolic activity (e.g., mitochondrial reduction of resazurin), ciliary function (e.g., cilia beat frequency and average active area measurements), oxidative stress (e.g., measurement of reactive oxygen species), and the release of pro-inflammatory markers (e.g., interleukin-6 and 8, and tumor necrosis factor-α). MucilAir is also amenable to histological analysis (e.g., using light or electron microscopy), providing visual observations that can be directly compared to local effects in human tissues. Furthermore, the generated results can be quantitative, circumventing subjectivity in measurements.

## 3 Application of the RHRE, MucilAir, to assess substance-mediated respiratory effects

### 3.1 General considerations

#### 3.1.1 Chemical domain

MucilAir has been widely applied across multiple research areas, including chemical and pharmaceutical toxicity testing, disease modeling (Beyeler et al., 2018; Phan et al., 2023), and respiratory infection studies (Hvorecny et al., 2018; Essaidi-Laziosi et al., 2020; Pizzorno et al., 2020). This RHRE has supported assessments of a broad range of substances, including agrochemicals (McGee Hargrove et al., 2021), fine and ultrafine particles (Despréaux et al., 2023), gasoline emissions (Cervena et al., 2019; Rossner et al., 2019; Sima et al., 2022), tobacco products (Iskandar, 2017), pharmaceuticals (De Servi et al., 2017; Balogh Sivars et al., 2018; Metz et al., 2018; Piqué and De Servi, 2018), and industrial chemicals (Sauer et al., 2013; Welch et al., 2021; Sharma et al., 2023; de Ávila et al., 2025; Sadekar et al., 2025) (see summary in Table 3).

**TABLE 3:** Summary of the potential applications of the RHRE, MucilAir.

Descriptor	Examples	Significance	Considerations
Test substance	Consumer goods (e.g., particles, fragrance ingredients, or tobacco products), gasoline emissions, industrial chemicals (e.g., solvents or insolation fibers), infection agents (e.g., viruses or bacteria), and pharmaceuticals	Any chemical class or insult that does not adversely interact with culture plastics can be tested. Commercially available exposure equipment is available, enabling the use of substances of different natures	A substance’s physicochemical properties should be considered when assessing the suitability of the test method
Modes of exposure	Liquid, aerosol (liquid and solid), gas	Amenable to liquid application, via pipetting, and aerosol or gas exposure using ALI exposure devices	Selecting the mode of exposure is linked to the substance’s physicochemical properties. For example, highly reactive substances or volatile chemicals may favor ALI exposure while liquid application may be preferred for less volatile, soluble, or difficult to aerosolize substances
Particle size	From nanometer to micrometer size	Can be used for a variety of material sizes, including real life heterogeneous mixtures, enabling the testing of larger particles than conventional *in vivo* animal tests	Testing will be most relevant when using a RHRE representing the target respiratory region. For example, nasal or tracheobronchial RHRE will not be as relevant if toxicity is only expected in the lower respiratory tract (where an alveolar RHRE may be more suitable)
Dosing and exposure time	Single and repeat dose, exposure time can be adapted to simulate different exposure conditions. Can include recovery period in between exposures or post-exposure	Allows generation of dose response curves, replicating both acute and chronic toxicities	Most studies currently available use relatively short repeat exposure protocols (less than 1 month). To date, repeat dosing protocols and exposure over longer periods are less common
Potential uses	Demonstrated usefulness for studying point of contact respiratory tissue irritation and mucociliary clearance, for example,	Reproduces adverse outcomes and endpoints of pharmacological interest	The cell types present should be considered when selecting an appropriate model system. For example, the model has not yet been demonstrated to have utility for studying respiratory sensitization or bronchoconstriction (asthma)

Substances in diverse physical forms can be tested using MucilAir, including solids (e.g., particles (Frieke Kuper et al., 2015; Kooter et al., 2017; Dankers et al., 2018; George et al., 2019), fibers (Chortarea et al., 2017; Beyeler et al., 2018; Hufnagel et al., 2021)), and liquid aerosols (Ishikawa et al., 2018; Bishop et al., 2022; Viegas et al., 2024; de Ávila et al., 2025), gases (Sharma et al., 2023), and vapors (Anderson et al., 2013; Moreau et al., 2022; Paudel et al., 2023; Sharma et al., 2023). The model has also been used to test a wide size range of solid materials, from the nanometer (Chortarea et al., 2017; Hufnagel et al., 2021) to micrometer size (Metz et al., 2018; Donkers et al., 2022), offering increased human relevance, since testing the larger of these particles would be restricted by the smaller size of the rat airways (Stucki et al., 2024).

Nasal and tracheobronchial RHRE (Jackson et al., 2018; Hwang et al., 2021; Wallace et al., 2023; Choe et al., 2026), including MucilAir (Balogh Sivars et al., 2018; de Ávila et al., 2025; Sadekar et al., 2025), have been used in a number of studies to assess diverse chemistries. A systematic evaluation of the suitability of each model to test specific chemistries could support further harmonization of testing protocols (e.g., exposure times, modes of exposure, or single versus repeated dosing), be adapted to different chemistries as needed, and aid in the ability to more directly compare data. Such harmonized protocols and multi-laboratory validation studies are developing and will further clarify the strengths and limitations of specific RHRE models and RHRE-based methods.

#### 3.1.2 Mode of exposure

In addition to the assessment of various types of substances, MucilAir has been shown to be useful for testing via multiple modes of exposure (e.g., liquid pipetting or aerosol). Depending on the purpose of testing and nature of the test article, substances can be delivered in small liquid quantities (via pipetting) (Sauer et al., 2013; Balogh Sivars et al., 2018; McGee Hargrove et al., 2021; Welch et al., 2021) or using *in vitro* ALI exposure devices that allow for realistic exposure to an airborne substance (Metz et al., 2018; Mistry et al., 2020; Oldham et al., 2020; Petersen et al., 2021; Sharma et al., 2023).

The mode of application for a test substance is one consideration, and the advantages and disadvantages of liquid application versus specialized ALI exposure devices have been discussed elsewhere (Wallace et al., 2025). Ease of use, dosimetry, and method transferability are typical factors in favor of liquid application, whereas the use of sophisticated aerosol systems offers physiological relevance and requires specialized expertise (e.g., aerosol engineers). A substance’s physicochemical properties will guide the selection of exposure mode. For example, highly hydrolyzing substances may degrade quickly when diluted in aqueous solutions, and exposure as non-humidified aerosols or vapors is preferrable (e.g., silanes (Sharma et al., 2023)). Liquid application may be preferred for difficult to aerosolize substances. Generally, studies have demonstrated value in different modes of exposure depending on the testing scenario and purpose, and as more data are generated, it will be useful to conduct a systematic comparison that readily highlights the value of each mode under specific conditions.

#### 3.1.3 Exposure duration

To replicate the varied exposure scenarios that humans may encounter, MucilAir is amenable to different exposure durations in either single or repeat dosing protocols. Exposure duration can be adapted, with studies ranging from a few hours to several days. MucilAir’s long-term stability (Baxter et al., 2015; Hoffmann et al., 2018), enables the evaluation of responses to acute (McGee Hargrove et al., 2021; Welch et al., 2021; Sharma et al., 2023; Boisleve et al., 2025; Sadekar et al., 2025) or repeated exposures (Balogh Sivars et al., 2018; Cervena et al., 2019; Rossner et al., 2021; Tratnjek et al., 2021; Sima et al., 2022; de Ávila et al., 2025).

#### 3.1.4 Range of effects

Severity of effects can be assessed, as dose-dependent responses can be observed when exposed to varying concentrations of a test substance, allowing for the differentiation of more or less toxic substances (Balogh Sivars et al., 2018; Moreau et al., 2022; de Ávila et al., 2025; Sadekar et al., 2025). After exposure, MucilAir tissues can be directly processed or maintained in culture using different recovery windows, thus providing insights on the reversibility of toxicity-induced effects over time (Welch et al., 2021; Sharma et al., 2023).

Current data streams support the use of RHRE models to identify toxic substances that cause point of contact toxicity (Jackson et al., 2018; McGee Hargrove et al., 2021; Choe et al., 2026). Some *in vitro* RHRE studies demonstrate differences in chemical toxicities when compared to *in vivo* rat tests; however, it is not yet clear which may be more relevant to human outcomes or whether the study designs were similarly optimized to enable such direct comparison of results (Jackson et al., 2018; Wallace et al., 2023).

Overall, a proper experimental design (e.g., airflow, volume for liquid exposure, number and timing of washes, and exposure duration) needs to be carefully developed to ensure the data obtained is physiologically relevant and fit for its intended purpose (Mallek et al., 2023; Roper and Neill, 2023; Recio et al., 2025), and should be informed by a substance’s physicochemical properties. RHRE-based methods can also be combined with other *in vitro*, *in chemico*, and *in silico* data to provide additional information, and the testing approach used will depend on the testing question (McGee Hargrove et al., 2021; Boisleve et al., 2025; de Ávila et al., 2025; Sadekar et al., 2025).

### 3.2 Use of RHRE-based methods for regulatory applications

To ensure a test method is fit for purpose, the goal of the testing must be considered. In the following section, we highlight two case studies that used MucilAir to address a regulatory need within the U.S. and European jurisdictions, demonstrating the fit-for-purpose application of an RHRE-based test method within a regulatory context (US EPA, 2021; SCCS, 2024).

For each of these case studies, consideration was given to selecting the appropriate model system to expose to the test substance. The area of the respiratory tract reached by the test substance was predicted using computational models, such as computational fluid dynamic modeling (Case Study 1), or by inferring from the physicochemical properties of the test substance and the intended use of the consumer product containing it (Case Study 2). In both cases, consideration of human relevance led to testing in the RHRE, MucilAir. Consideration was also given to human-relevant exposure levels, which can be estimated through monitoring, for example, during occupational use of the product. A common thread to the following case studies is a testing strategy underpinned by a mechanistic understanding of toxicity in humans that informed the study design and endpoints assessed. The data were interpreted, for example, by extrapolation of points of departure from the *in vitro* data using the US Environmental Protection Agency (EPA)’s Benchmark Dose (BMD) tools (https://www.epa.gov/bmds) and subsequent *in vitro* to *in vivo* extrapolation (IVIVE) to derive human equivalent concentrations (Case Study 1). Alternatively, deterministic modeling or comparison to the Toxicological Thresholds of Concern (TTC) were used to estimate margins of safety (Case Study 2). Ultimately, the test results were presented to regulators and/or a designated expert committee to ensure transparency, data integrity, and opportunity for thorough review. The goal was to present a comprehensive and scientifically sound data package that provided risk assessors with the necessary information for confident decision making.

#### 3.2.1 Case study 1: use of an RHRE in the re-registration of a fungicide

In response to a US EPA request for the re-evaluation of inhalation toxicity of the pesticide chlorothalonil, a non-animal weight of evidence approach was proposed *in lieu* of a 90-day rat inhalation study. The sponsors sought to use an approach that was reliable and human-relevant without the associated ethical concerns of testing an irritating chemical in animals.

Since there was no regulatory precedent, effective communication and scientific evaluation by regulators and experts helped shape the final approach and risk assessment. The approach included a combination of information about the mechanism of action of chlorothalonil, expected human exposure, *in silico* modelling, *in vitro* testing, and IVIVE to derive a human equivalent concentration for risk assessment. Computational fluid dynamics (CFD), particle size distribution, and operator breathing measurements were performed. Using the CFD modeling results, the upper conducting airways were identified as a primary site of particle deposition, supporting the use of MucilAir for *in vitro* testing.

MucilAir was selected for *in vitro* assessments due to its human relevance and ability to assess contact irritants (Huang et al., 2011; Balogh Sivars et al., 2018). The biological endpoints assessed in the RHRE-based approach were based on their predictivity of *in vivo* irritation (Balogh Sivars et al., 2018; OECD, 2022) and included cytotoxicity, barrier function, and metabolic activity, which are relevant to the mechanism of action of chlorothalonil and the adverse outcome of interest.

In brief, MucilAir tissues, single and pooled donors, were exposed by pipetting (30 µL/well, ∼91 μL/cm^2^) serial dilutions of the fungicide for 8- and 24-h single exposures and repeated 24-h exposures for 5 days. BMD modeling was used for each endpoint to derive inhalation points of departure. The BMD values for the most sensitive endpoint with the better model fits, resazurin (cytotoxicity), were selected to calculate human equivalent concentrations and doses (US EPA, 2021). The BMD results were used in conjunction with site-specific CFD data to calculate human equivalent concentrations and doses for different durations and particle size distributions corresponding to those anticipated for occupational and non-occupational scenarios (US EPA, 2021).

A preliminary risk assessment using single donors and single 8-h and 24-h exposures was first presented to a Federal Insecticide, Fungicide, and Rodenticide Act (FIFRA) Scientific Advisory Panel (SAP) in 2018. The SAP final report (EPA, 2019) supported the use of the approach for contact irritants and provided recommendations to improve and further support the approach. In particular, the SAP recommended collecting additional information on the impact of repeat dosing on the *in vitro* measurements, consideration of clearance, and differences in nasal and oral breathing deposition. The sponsor then repeated the study including a 5-day repeated study and the use of pooled donors. Generally, the single 24-h exposure resulted in lower BMD values than the single 8-h exposure and there were no substantial differences in the single and 5-day exposure scenarios.

The approach and results were included in the EPA draft risk assessment (US EPA, 2021), published in peer-reviewed journals (Corley et al., 2021; McGee Hargrove et al., 2021; Ramanarayanan et al., 2022), and as an OECD Integrated Approach for Testing and Assessment (IATA) case study (OECD, 2022).

This case study presented a first-of-its-kind example where a RHRE-based approach was applied in a regulatory setting to conduct an inhalation risk assessment of an agrochemical in place of an *in vivo* test.

The use of human cells and reproducible, quantitative outcomes limited uncertainty and demonstrated the value of using human cell-based testing approaches.

#### 3.2.2 Case study 2: use of an RHRE to evaluate the potential inhalation toxicity of a cosmetic ingredient

Acetylated Vetiver Oil (AVO) is a naturally derived fragrance ingredient, composed of more than 130 constituents and commonly used in cosmetic products (SCCS, 2019). The European Commission requested the EU Scientific Committee on Consumer Safety (SCCS) to carry out a safety assessment on AVO (SCCS, 2019). This assessment revealed no concern for the use of AVO in leave-on and rinse-off cosmetic products at the concentrations typically used in cosmetic products. However, its assessment did not extend to spray products, which may present inhalation concerns. To assess the potential systemic and irritating local effects resulting from inhalation of spray products containing AVO, the sponsor applied a non-animal, weight-of-evidence approach, in line with the European Union’s ban on animal testing for cosmetic ingredients.

Given AVO’s low volatility (vapor pressure is 0.01–0.1 Pa at 20 °C) (ECHA, 2019), inhalation exposure following rapid volatilization was considered negligible. Systemic exposure via the inhalation and dermal routes was calculated for the sprayed fraction using a deterministic 2-Box model, which assumes the emitted material is homogeneously dispersed in both short-term near field and longer, far field environments (Box A and Box B) (Steiling et al., 2014). The calculations were conservative, assuming worst-case conditions for spray products under typical consumer use conditions. Across product types, the calculated systemic exposure to AVO via the inhalation route was much lower than via the dermal route, minimally contributing to the prior dermal calculations. The calculated total systemic exposure via the dermal and inhalation routes was compared to the no observed adverse effect level (NOAEL) for systemic toxicity, resulting in a margin of safety indicative of no concern of systemic toxicity.

Local respiratory toxicity was also evaluated following the inhalation threshold of toxicological concern (TTC_inh_) and an *in vitro* local respiratory irritation test using the RHRE, MucilAir (Boisleve et al., 2025). The TTC approach revealed no concern, as all typical concentrations of AVO in cosmetics were well below the TTC_inh_ for local respiratory effects. The experimental plan used a direct liquid application approach using a small volume (10 µL), representing a worst-case scenario for acute exposure (24 h). The study included endpoints to assess barrier integrity (TEER), cytotoxicity (LDH), cytokine release, and histopathology. The concentrations used in the study were determined based on the solubility of the expected nominal maximal dose of AVO in different solvents and the impact of different doses on the viability and integrity of MucilAir based on a dose range finding study. The highest concentration tested for all endpoints, 5% (w/w), caused minor effects indicative of toxicity, which reversed 7 days post-exposure. A NOAEL of 1% (w/w) was calculated as the point of departure based on the data. Notably, however, the higher concentrations tested were an order of magnitude higher than exposures to the respiratory tract upon use of cosmetic products. Therefore, the effects observed at higher concentrations were not deemed indicative of consumer risk.

The study findings were published (Boisleve et al., 2025) and independently reviewed by the SCCS (SCCS, 2024). The SCCS concluded that AVO is safe for use in cosmetics up to 0.9% (w/w) in fragrance pump sprays, 0.05% (w/w) in deodorant sprays, and 0.1% (w/w) in hairsprays and body lotion sprays (SCCS, 2024). Following the SCCS opinion documents and recommendations, the EU Commission issued Commission Regulation (EU) 2026/909. The Commission followed the SCCS recommendation and concluded that “the use of acetylated vetiver oil in cosmetic products should be restricted to the maximum concentrations proposed by the SCCS” (European Commission, 2026).

This case study highlighted the utility of RHRE in evaluating the local respiratory irritation potential of low volatility, low concentration fragrance ingredients in sprayable cosmetics and of complex composition. The data were useful in risk assessment and decision-making, and the approach set a precedent for how similar fragrance materials in spray products can be assessed.

## 4 Discussion

Human-relevant *in vitro* test methods are being developed and applied in regulatory decision making. These methods circumvent species uncertainties, can efficiently produce quantitative and qualitative information, provide mechanistic insights, and can operate in higher throughput. For adoption in regulated environments, a test method needs to demonstrate regulatory readiness, which can be assessed by evaluating its fitness for purpose, human biological relevance, technical characterization, data integrity and transparency, and independent review (van der Zalm et al., 2022; ICCVAM, 2024).

This review demonstrates the RHRE model, MucilAir, is biologically relevant to understanding human effects and how it can be applied in different contexts of use. Upper airway RHRE models, including MucilAir, are structurally similar to human respiratory epithelium and comprise the three epithelial cell types found in the nasal, tracheal, and bronchial regions (Wallace et al., 2025). Functionally, MucilAir replicates key elements of the respiratory epithelium: it produces a tight barrier with selective permeability to compounds, produces mucus, has beating cilia, is metabolically active, and responds to stimuli similarly to human respiratory epithelium. It also demonstrates histological changes that can be directly compared to human clinical and epidemiological observations of respiratory irritation.

Like any model, whether to use a RHRE model will depend on the chemical substance, purpose of testing, and context of use. For example, MucilAir does not contain immune, vascular, or smooth muscle cells and, therefore, is not intended to be used to investigate certain adverse responses. For example, it is not well suited to investigate immune-mediated responses, including respiratory sensitization or bronchoconstriction. While MucilAir responds to proinflammatory triggers, the lack of an immune component limits its response to substances acting through proinflammatory signaling (e.g., lipopolysaccharide (de Ávila et al., 2025)), for which very high doses need to be applied to elicit measurable responses. Further, few studies have looked at MucilAir’s permeability and transport of substances (Reus et al., 2014; Mercier et al., 2019), and expanding the number of investigated substances would help in better understanding the model’s capabilities in this area. Additionally, while research is ongoing to further characterize the metabolic competence of MucilAir and its suitability to test substances requiring specific metabolic activation, published data demonstrate its similarity to human respiratory epithelium and ability to generate human-relevant data for multiple toxicity endpoints. Lastly, RHRE represent one region of the respiratory tract and as such cannot be used to assess general systemic toxicity; however, they may prove valuable in an integrated approach to assess systemic toxicity.

MucilAir’s human relevance and technical maturity have led to its use in supporting safety decisions (US EPA, 2021; SCCS, 2024). The case studies presented in this paper provide real-world examples showing how information from multiple data streams (e.g., chemical properties, human exposure scenarios, and region of the respiratory tract likely to be exposed) can be used to inform the selection of the appropriate model system and generate qualitative and quantitative data for risk assessment that can predict effects of inhalation exposure in humans.

RHRE have been used for more than two decades, and there is now a focus on standardizing test method protocols to facilitate comparison of data across studies and assessment of method robustness.

For example, an RHRE-based method for assessing portal-of-entry effects, developed using protocols comparable to those in the case studies presented here, has undergone a multi-laboratory, multi-country study. Further, intra- and inter-laboratory studies, and international cross-sector efforts have contributed to the development of reporting standards and minimum requirements for measurement and standardization of relevant endpoints (TEER and CBF) (Reus et al., 2014; Hoffmann et al., 2018; Behrsing et al., 2022; Sharma et al., 2025). Additional efforts are underway to help better understand other factors, such as the contribution of mucus production, donor selection, and exposure set-up. This is important because, for example, the thoroughness and time of cell washing can influence the presence of mucus and, therefore, the contact of the test substance with the cells. Further, information from single or pooled donors can be useful, and an understanding of the advantages and limitations of each, as well as the goals of the study, can help in model selection. Finally, case studies found in the published literature as well as ongoing work are helping in making decisions about exposure modes, and whether a simpler liquid dosing application is sufficient or more physiological exposure systems add value.

Overall, this paper reviews the biological relevance of RHRE, particularly MucilAir. To move from promising case studies to routine regulatory application, test methods based on RHRE will also need to be evaluated for their technical characterization and fitness for purpose. This involves harmonizing protocols, testing a variety of chemicals in multiple laboratories, and developing robust acceptability criteria for these methods. Ultimately, complementary test methods can be used assess different chemistries, modes of exposure or adverse outcomes, providing a comprehensive, powerful toolkit for assessing inhalation effects.

## References

[B1] Anderson S. E. Khurshid S. S. Meade B. J. Lukomska E. Wells J. R. (2013). Toxicological analysis of limonene reaction products using an *in vitro* exposure system. Toxicol. Vitro 27 (2), 721–730. 10.1016/j.tiv.2012.11.017 23220291 PMC4680979

[B2] Baiocco G. George I. Garcia-Argote S. Guardamagna I. Lonati L. Lamartinière Y. (2021). A 3D *in vitro* model of the human airway epithelium exposed to tritiated water: Dosimetric estimate and cytotoxic effects. Radiat. Res. 195 (3), 265–274. 10.1667/RADE-20-00208.1 33400793

[B3] Balogh Sivars K. Sivars U. Hornberg E. Zhang H. Brändén L. Bonfante R. (2018). A 3D human airway model enables prediction of respiratory toxicity of inhaled drugs *in vitro* . Toxicol. Sci. 162 (1), 301–308. 10.1093/toxsci/kfx255 29182718

[B4] Baxter A. Thain S. Banerjee A. Haswell L. Parmar A. Phillips G. (2015). Targeted omics analyses, and metabolic enzyme activity assays demonstrate maintenance of key mucociliary characteristics in long term cultures of reconstituted human airway epithelia. Toxicol. Vitro 29 (5), 864–875. 10.1016/J.TIV.2015.03.004 25863282

[B5] Behrsing H. P. Wahab A. Ukishima L. Grodi C. Frentzel S. Johne S. (2022). Ciliary beat frequency: proceedings and recommendations from a multi-laboratory ring trial using 3-D reconstituted human airway epithelium to model mucociliary clearance. Altern. Laboratory Animals ATLA 50 (4), 263–309. 10.1177/02611929221114383 35938181

[B7] Beyeler S. Chortarea S. Rothen-Rutishauser B. Petri-Fink A. Wick P. Tschanz S. A. (2018). Acute effects of multi-walled carbon nanotubes on primary bronchial epithelial cells from COPD patients. Nanotoxicology 12 (7), 699–711. 10.1080/17435390.2018.1472310 29804489

[B8] Bishop E. Terry A. East N. Breheny D. Gaça M. Thorne D. (2022). A 3D *in vitro* comparison of two undiluted e-cigarette aerosol generating systems. Toxicol. Lett. 358, 69–79. 10.1016/j.toxlet.2022.01.002 35032609

[B9] Boisleve F. Vandecasteele H. A. Baily J. Czuchrowski K. Diop M. Gadras C. (2025). Use of NAMs in a weight of evidence approach to evaluate the safety via the inhalation route of acetylated vetiver oil, in spray products. Regul. Toxicol. Pharmacol. 162 (November), 105905. 10.1016/j.yrtph.2025.105905 40639679

[B10] Bovard D. Sandoz A. Luettich K. Frentzel S. Iskandar A. Marescotti D. (2018). A lung/liver-on-a-chip platform for acute and chronic toxicity studies. Lab a Chip 18 (24), 3814–3829. 10.1039/C8LC01029C 30460365

[B11] Bovard D. Giralt A. Trivedi K. Neau L. Kanellos P. Iskandar A. (2020). Comparison of the basic morphology and function of 3D lung epithelial cultures derived from several donors. Curr. Res. Toxicol. 1, 56–69. 10.1016/j.crtox.2020.08.002 34345837 PMC8320645

[B12] Caulfuty M. Huang S. (2009). A novel *in vitro* cell model of the human airway epithelium. 3R Info Bull. 41 (41), 1–2. Available online at: https://www.forschung3r.ch/data/publications/Huang-Bul41.pdf (Accessed December 5, 2025).

[B13] Cervena T. Vrbova K. Rossnerova A. Topinka J. Rossner P. (2019). Short-term and long-term exposure of the MucilAir^TM^ model to polycyclic aromatic hydrocarbons. ATLA Altern. Laboratory Animals 47 (1), 9–18. 10.1177/0261192919841484 31237164

[B14] Chapman F. Pour S. J. Wieczorek R. Trelles Sticken E. Budde J. Röwer K. (2023). Twenty-eight day repeated exposure of human 3D bronchial epithelial model to heated tobacco aerosols indicates decreased toxicological responses compared to cigarette smoke. Front. Toxicol. 5, 1076752. 10.3389/FTOX.2023.1076752 36875887 PMC9979258

[B15] Choe J. W. Lee G. H. Lee S. H. Kim G. E. Kim H. R. Lim K. M. (2026). Development of an *in vitro* acute respiratory toxicity test using the SoluAirway^TM^, 3D human airway model. Toxicol. Vitro 111, 106175. 10.1016/j.tiv.2025.106175 41260537

[B16] Chortarea S. Barosova H. Clift M. J. D. Wick P. Petri-Fink A. Rothen-Rutishauser B. (2017). Human asthmatic bronchial cells are more susceptible to subchronic repeated exposures of aerosolized carbon nanotubes at occupationally relevant doses than healthy cells. ACS Nano 11 (8), 7615–7625. 10.1021/acsnano.7b01992 28505409

[B17] Clippinger A. J. Allen D. Jarabek A. M. Corvaro M. Gaça M. Gehen S. (2018a). Alternative approaches for acute inhalation toxicity testing to address global regulatory and non-regulatory data requirements: an international workshop report. Toxicol. Vitro 48, 53–70. 10.1016/j.tiv.2017.12.011 29277654 PMC8215693

[B18] Clippinger A. J. Allen D. Behrsing H. BéruBé K. A. Bolger M. B. Casey W. (2018b). Pathway-based predictive approaches for non-animal assessment of acute inhalation toxicity. Toxicol Vitro 52, 131–145. 10.1016/j.tiv.2018.06.009 29908304 PMC6760245

[B19] Corley R. A. Kuprat A. P. Suffield S. R. Kabilan S. Hinderliter P. M. Yugulis K. (2021). New approach methodology for assessing inhalation risks of a contact respiratory cytotoxicant: computational fluid dynamics-based aerosol dosimetry modeling for cross-species and *in vitro* comparisons. Toxicol. Sci. 182 (2), 243–259. 10.1093/toxsci/kfab062 34077545 PMC8331159

[B20] Czekala L. Wieczorek R. Simms L. Yu F. Budde J. Trelles Sticken E. (2021). Multi-endpoint analysis of human 3D airway epithelium following repeated exposure to whole electronic vapor product aerosol or cigarette smoke. Curr. Res. Toxicol. 2, 99–115. 10.1016/J.CRTOX.2021.02.004 34345855 PMC8320624

[B21] Dankers A. C. A. Kuper C. F. Boumeester A. J. Fabriek B. O. Kooter I. M. Gröllers‐Mulderij M. (2018). A practical approach to assess inhalation toxicity of metal oxide nanoparticles *in vitro* . J. Appl. Toxicol. 38 (2), 160–171. 10.1002/jat.3518 28960351

[B22] de Ávila R. I. Müller I. Barlow H. Middleton A. M. Theiventhran M. Basili D. (2025). Evaluation of a non-animal toolbox informed by adverse outcome pathways for human inhalation safety. Front. Toxicol. 7 (February), 1–30. 10.3389/ftox.2025.1426132 PMC11885506 40061084

[B23] De Servi B. Ranzini F. Piqué N. (2017). Protective barrier properties of rhinosectan® spray (containing xyloglucan) on an organotypic 3D airway tissue model (MucilAir): results of an *in vitro* study. Allergy, Asthma and Clin. Immunol. 13 (1), 37. 10.1186/s13223-017-0209-6 28811823 PMC5553660

[B24] Despréaux P. Jeanton C. Desaulle D. Al Zallouha M. Verdin A. Momas I. (2023). Innovative graph analysis method to assess gene expression modulation after fine particles exposures of 3D human airway epithelia. Environ. Res. 221, 115296. 10.1016/j.envres.2023.115296 36642119

[B25] Donkers J. M. Höppener E. M. Grigoriev I. Will L. Melgert B. N. van der Zaan B. (2022). Advanced epithelial lung and gut barrier models demonstrate passage of microplastic particles. Microplastics Nanoplastics 2 (1), 6. 10.1186/s43591-021-00024-w

[B26] ECHA (2019). Dossier on Vetiveria zizanioides, ext., acetylated. Available online at: https://echa.europa.eu/registration-dossier/-/registered-dossier/22147/4/7 (Accessed September 4, 2025).

[B103] EPA (2019). FIFRA Scientific Advisory Panel Meeting Minutes and Final Report No. 2019-01 Peer Review on Evaluation of a Proposed Approach to Refine the Inhalation Risk Assessment for Point of Contact Toxicity: A Case Study Using a New Approach Methodology (NAM) December 4 and 6, 2018 FIFRA Scientific Advisory Panel Meeting. Available online at: https://www.regulations.gov/document/EPA-HQ-OPP-2018-0517-0030 (Accessed April 27, 2026).

[B27] Essaidi-Laziosi M. Geiser J. Huang S. Constant S. Kaiser L. Tapparel C. (2020). Interferon-dependent and respiratory virus-specific interference in dual infections of airway epithelia. Sci. Rep. 10 (1), 1–10. 10.1038/s41598-020-66748-6 32581261 PMC7314816

[B28] Esteban Enjuto L. Taty Poaty V. Bouveret M. Song H. Constant S. Patarin J. (2024). Rheological comparison of sputum and reconstituted airway epithelium mucus. Sci. Rep. 14 (1), 1–8. 10.1038/s41598-024-80932-y 39738355 PMC11685952

[B29] Frieke Kuper C. Gröllers-Mulderij M. Maarschalkerweerd T. Meulendijks N. M. M. Reus A. van Acker F. (2015). Toxicity assessment of aggregated/agglomerated cerium oxide nanoparticles in an *in vitro* 3D airway model: the influence of mucociliary clearance. Toxicol. Vitro 29 (2), 389–397. 10.1016/j.tiv.2014.10.017 25448805

[B30] George I. Uboldi C. Bernard E. Sobrido M. S. Dine S. Hagège A. (2019). Toxicological assessment of ITER-Like tungsten nanoparticles using an *in vitro* 3D human airway epithelium model. Nanomaterials 9 (10), 1374. 10.3390/nano9101374 31557883 PMC6836029

[B31] Hervé P. Nathalie C. Gaëlle D. Margueritta A. Z. Sophie A. Valérie D. (2024). An original device to assess the respiratory impact of indoor air VOCs mixture using an *in vitro* approach. Indoor Environ. 1 (3), 100037. 10.1016/j.indenv.2024.100037

[B32] Hoffmann W. Gradinaru J. Farcal L. Caul-Futy M. Huang S. Wiszniewski L. (2018). Establishment of a human 3D tissue-based assay for upper respiratory tract absorption. Appl. Vitro Toxicol. 4 (2), 139–148. 10.1089/AIVT.2017.0035/ASSET/IMAGES/LARGE/FIGURE5.JPEG

[B33] Huang S. Wiszniewski L. Constant S. (2011). “The use of *in vitro* 3D cell models in drug development for respiratory diseases,” in Drug Discovery and Development - Present and Future (London, United Kingdom: InTechOpen), 169–190. 10.5772/28132

[B34] Hufnagel M. May N. Wall J. Wingert N. Garcia-Käufer M. Arif A. (2021). Impact of nanocomposite combustion aerosols on a549 cells and a 3d airway model. Nanomaterials 11 (7), 1685. 10.3390/nano11071685 34199005 PMC8304990

[B35] Huh D. Matthews B. D. Mammoto A. Montoya-Zavala M. Hsin H. Y. Ingber D. E. (2010). Reconstituting organ-level lung functions on a chip. Science 328 (5986), 1662–1668. 10.1126/science.1188302 20576885 PMC8335790

[B36] Hvorecny K. L. Dolben E. Moreau-Marquis S. Hampton T. H. Shabaneh T. B. Flitter B. A. (2018). An epoxide hydrolase secreted by *Pseudomonas aeruginosa* decreases mucociliary transport and hinders bacterial clearance from the lung. Am. J. Physiology - Lung Cell. Mol. Physiology 314 (1), L150–L156. 10.1152/ajplung.00383.2017 28982736 PMC5866430

[B37] Hwang J. hyun Jeong H. Jung Y. on Nam K. T. Lim K. M. (2021). Skin irritation and inhalation toxicity of biocides evaluated with reconstructed human epidermis and airway models. Food Chem. Toxicol. 150, 112064. 10.1016/j.fct.2021.112064 33596452

[B38] ICCVAM. (2024). Validation, qualification, and regulatory acceptance of new approach methodologies. 10.224277/NICEATM-2 40418713

[B39] Ishikawa S. Matsumura K. Kitamura N. Ishimori K. Takanami Y. Ito S. (2018). Application of a direct aerosol exposure system for the assessment of biological effects of cigarette smoke and novel tobacco product vapor on human bronchial epithelial cultures. Regul. Toxicol. Pharmacol. 96, 85–93. 10.1016/j.yrtph.2018.05.004 29730447

[B40] Iskandar A. R. Martin F. Talikka M. Schlage W. K. Kostadinova R. Mathis C. (2013). Systems approaches evaluating the perturbation of xenobiotic metabolism in response to cigarette smoke exposure in nasal and bronchial tissues. BioMed Res. Int. 2013 (1), 1–14. 10.1155/2013/512086 24224167 PMC3808713

[B41] Iskandar A. Mathis C. Martin F. Leroy P. Sewer A. Majeed S. (2017). 3-D nasal cultures: systems toxicological assessment of a candidate modified-risk tobacco product. ALTEX 34, 23–48. 10.14573/altex.1605041 27388676

[B42] Jackson G. R. Maione A. G. Klausner M. Hayden P. J. (2018). Prevalidation of an acute inhalation toxicity test using the EpiAirway *in vitro* human airway model. Appl. Vitro Toxicol. 4 (2), 149–158. 10.1089/aivt.2018.0004 29904643 PMC5994905

[B43] Kelty J. Kovalchuk N. Uwimana E. Yin L. Ding X. Van Winkle L. (2022). *In vitro* airway models from mice, rhesus macaques, and humans maintain species differences in xenobiotic metabolism and cellular responses to naphthalene. Am. J. Physiology - Lung Cell. Mol. Physiology 323 (3), L308–L328. 10.1152/ajplung.00349.2021 35853015 PMC9423729

[B44] Kim J. W. Jeong M. H. Kim G. E. Han Y. B. Park Y. J. Chung K. H. (2022). Comparison of 3D airway models for the assessment of fibrogenic chemicals. Toxicol. Lett. 356, 100–109. 10.1016/j.toxlet.2021.12.007 34902520

[B45] Kooter I. M. Gröllers-Mulderij M. Duistermaat E. Kuper F. Schoen E. D. (2017). Factors of concern in a human 3D cellular airway model exposed to aerosols of nanoparticles. Toxicol. Vitro 44 (July), 339–348. 10.1016/j.tiv.2017.07.006 28705761

[B46] Lacroix G. Koch W. Ritter D. Gutleb A. C. Larsen S. T. Loret T. (2018). Air-liquid interface *in vitro* models for respiratory toxicology research: consensus workshop and recommendations. Appl. Vitro Toxicol. 4 (2), 91–106. 10.1089/aivt.2017.0034 32953944 PMC7500038

[B47] Ladics G. S. Price O. Kelkar S. Herkimer S. Anderson S. (2021). A weight-of-the-evidence approach for evaluating, *in lieu* of animal studies, the potential of a novel polysaccharide polymer to produce lung overload. Chem. Res. Toxicol. 34 (6), 1430–1444. 10.1021/acs.chemrestox.0c00301 33881304

[B101] Lee N. Jang D. Y. Lee D. H. Jeong H. Nam K. T. Choi D.-W. (2021). Local toxicity of biocides after direct and aerosol exposure on the human skin epidermis and airway tissue models. Toxics 9, 29. 10.3390/toxics9020029 33546295 PMC7913294

[B48] Lodes N. Seidensticker K. Perniss A. Nietzer S. Oberwinkler H. May T. (2020). Investigation on ciliary functionality of different airway epithelial cell lines in three-dimensional cell culture. Tissue Eng. - Part A 26 (7–8), 432–440. 10.1089/ten.tea.2019.0188 31696788 PMC7187987

[B49] Mallek N. M. Martin E. M. Dailey L. A. McCullough S. D. (2023). Liquid application dosing alters the physiology of air-liquid interface (ALI) primary human bronchial epithelial cell/lung fibroblast co-cultures and *in vitro* testing relevant endpoints. Front. Toxicol. 5 (January), 1–19. 10.3389/ftox.2023.1264331 PMC10922929 38464699

[B50] McDougall C. M. Blaylock M. G. Douglas J. G. Brooker R. J. Helms P. J. Walsh G. M. (2008). Nasal epithelial cells as surrogates for bronchial epithelial cells in airway inflammation studies. Am. J. Respir. Cell Mol. Biol. 39 (5), 560–568. 10.1165/rcmb.2007-0325OC 18483420 PMC2643208

[B51] McGee Hargrove M. Parr-Dobrzanski B. Li L. Constant S. Wallace J. Hinderliter P. (2021). Use of the MucilAir airway assay, a new approach methodology, for evaluating the safety and inhalation risk of agrochemicals. Appl. Vitro Toxicol. 7 (2), 50–60. 10.1089/aivt.2021.0005

[B52] Mercer R. R. Russell M. L. Roggli V. L. Crapo J. D. (1994). Cell number and distribution in human and rat airways. Am. J. Respir. Cell Mol. Biol. 10 (6), 613–624. 10.1165/ajrcmb.10.6.8003339 8003339

[B53] Mercier C. Jacqueroux E. He Z. Hodin S. Constant S. Perek N. (2019). Pharmacological characterization of the 3D MucilAir^TM^ nasal model. Eur. J. Pharm. Biopharm. 139, 186–196. 10.1016/j.ejpb.2019.04.002 30951820

[B54] Metz J. Knoth K. Groß H. Lehr C.-M. Stäbler C. Bock U. (2018). Combining MucilAir^TM^ and vitrocell® powder chamber for the *in vitro* evaluation of nasal ointments in the context of aerosolized pollen. Pharmaceutics 10 (2), 56. 10.3390/pharmaceutics10020056 29747472 PMC6027377

[B55] Mistry A. Bowen L. E. Dzierlenga M. W. Hartman J. K. Slattery S. D. (2020). Development of an *in vitro* approach to point-of-contact inhalation toxicity testing of volatile compounds, using organotypic culture and air-liquid interface exposure. Toxicol. Vitro 69, 104968. 10.1016/j.tiv.2020.104968 32805374

[B56] Moreau M. Fisher J. Andersen M. E. Barnwell A. Corzine S. Ranade A. (2022). NAM-Based prediction of point-of-contact toxicity in the lung: a case example with 1,3-dichloropropene. Toxicology 481 (July), 153340. 10.1016/j.tox.2022.153340 36183849

[B57] OECD (2022). “Case study on the use of an integrated approach for testing and assessment (IATA) for new approach methodology (NAM) for refining inhalation risk assessment from point of contact toxicity of the pesticide, chlorothalonil,” in Series of Testing and Assessment. No. 367 (Paris, France: OECD Publishing). Available online at: https://one.oecd.org/document/env/cbc/mono(2022)31 (Accessed September 4, 2025).

[B102] Oesch F. Fabian E. Landsiedel R. (2019). Xenobiotica-metabolizing enzymes in the lung of experimental animals, man and in human lung models. Arch. Toxicol. 93, 3419–3489. 10.1007/s00204-019-02602-7 31673725

[B58] Okuda K. Dang H. Kobayashi Y. Carraro G. Nakano S. Chen G. (2021). Secretory cells dominate airway CFTR expression and function in human airway superficial epithelia. Am. J. Respir. Crit. Care Med. 203 (10), 1275–1289. 10.1164/rccm.202008-3198OC 33321047 PMC8456462

[B59] Oldham M. J. Castro N. Zhang J. Lucci F. Kosachevsky P. Rostami A. A. (2020). Comparison of experimentally measured and computational fluid dynamic predicted deposition and deposition uniformity of monodisperse solid particles in the vitrocell® AMES 48 air-liquid-interface *in-vitro* exposure system. Toxicol. Vitro 67, 104870. 10.1016/j.tiv.2020.104870 32330563

[B60] Park S. Woo C. G. Cho Y.-J. (2024). Revolutionizing respiratory health research: “commercially-available lung-on-a-chip and air-liquid interface systems.”. Front. Lab a Chip Technol. 3, 1–8. 10.3389/frlct.2024.1373029

[B61] Patel V. Amin K. Allen D. Ukishima L. Wahab A. Grodi C. (2021). Comparison of long-term human precision-cut lung slice culture methodology and response to challenge: an argument for standardisation. Altern. Laboratory Animals 49 (5), 209–222. 10.1177/02611929211061884 34836458

[B62] Patel V. S. Amin K. Wahab A. Marimoutou M. Ukishima L. Alvarez J. (2023). Cryopreserved human precision-cut lung slices provide an immune competent pulmonary test system for “On-demand” use and long-term cultures. Toxicol. Sci. 191, 253–265. 10.1093/toxsci/kfac136 36617185 PMC9936202

[B63] Paudel I. Barutcu A. R. Samuel R. Moreau M. Slattery S. D. Scaglione J. (2023). Increasing confidence in new approach methodologies for inhalation risk assessment with multiple end point assays using 5-day repeated exposure to 1,3-dichloropropene. Toxicology 499, 153642. 10.1016/j.tox.2023.153642 37863466

[B64] Petersen E. J. Sharma M. Clippinger A. J. Gordon J. Katz A. Laux P. (2021). Use of cause-and-effect analysis to optimize the reliability of *in vitro* inhalation toxicity measurements using an air–liquid interface. Chem. Res. Toxicol. 34 (6), 1370–1385. 10.1021/acs.chemrestox.1c00080 34097823

[B65] Phan T. H. Shi H. Denes C. E. Cole A. J. Wang Y. Cheng Y. Y. (2023). Advanced pathophysiology mimicking lung models for accelerated drug discovery. Biomaterials Res. 27 (1), 1–21. 10.1186/s40824-023-00366-x 37098610 PMC10129441

[B66] Piqué N. De Servi B. (2018). Rhinosectan® spray (containing xyloglucan) on the ciliary function of the nasal respiratory epithelium; results of an *in vitro* study. Allergy, Asthma and Clin. Immunol. 14 (1), 41. 10.1186/s13223-018-0268-3 30337943 PMC6174573

[B67] Pizzorno A. Padey B. Julien T. Trouillet-Assant S. Traversier A. Errazuriz-Cerda E. (2020). Characterization and treatment of SARS-CoV-2 in nasal and bronchial human airway epithelia. Cell Rep. Med. 1 (4), 100059. 10.1016/j.xcrm.2020.100059 32835306 PMC7373044

[B68] Pohl M. O. Violaki K. Liu L. Gaggioli E. Glas I. von Kempis J. (2025). Comparative characterization of bronchial and nasal mucus reveals key determinants of influenza A virus inhibition. MSphere 10 (9), e00365-25. 10.1128/msphere.00365-25 40853004 PMC12482186

[B69] Ramanarayanan T. Szarka A. Flack S. Hinderliter P. Corley R. Charlton A. (2022). Application of a new approach method (NAM) for inhalation risk assessment. Regul. Toxicol. Pharmacol. 133, 105216. 10.1016/j.yrtph.2022.105216 35817205

[B70] Recio L. Samuel R. Elmore S. A. Scaglione J. (2025). Fifteen day repeat air: liquid interface air-only exposures can cause respiratory epithelium injury in MucilAir ^TM^ nasal respiratory epithelial cells that parallels chemically induced cytotoxicity. Toxicol. Mech. Methods 35 (1), 81–87. 10.1080/15376516.2024.2382794 39077774

[B71] Reus A. A. Maas W. J. M. Jansen H. T. Constant S. Staal Y. C. M. van Triel J. J. (2014). Feasibility of a 3D human airway epithelial model to study respiratory absorption. Toxicol. Vitro 28 (2), 258–264. 10.1016/j.tiv.2013.10.025 24216300

[B72] Roper C. S. Neill D. R. (2023). Transitioning acute *in vitro* inhalation toxicology testing to chronic and repeat dose testing; the challenge of mucus depletion in upper airway test systems and use of sputum mimics. Open Access J. Toxicol. 5 (4), 10–12. 10.19080/OAJT.2023.05.555669

[B73] Rossner P. Cervena T. Vojtisek-Lom M. Vrbova K. Ambroz A. Novakova Z. (2019). The biological effects of complete gasoline engine emissions exposure in a 3D human airway model (mucilair) and in human bronchial epithelial cells (BEAS-2B). Int. J. Mol. Sci. 20 (22), 5710. 10.3390/ijms20225710 31739528 PMC6888625

[B74] Rossner P. Cervena T. Vojtisek-Lom M. Neca J. Ciganek M. Vrbova K. (2021). Markers of lipid oxidation and inflammation in bronchial cells exposed to complete gasoline emissions and their organic extracts. Chemosphere 281, 130833. 10.1016/j.chemosphere.2021.130833 34015653

[B75] Roth D. Şahin A. T. Ling F. Tepho N. Senger C. N. Quiroz E. J. (2025). Structure and function relationships of mucociliary clearance in human and rat airways. Nat. Commun. 16 (1), 2446. 10.1038/s41467-025-57667-z 40069153 PMC11897160

[B76] Rotoli B. Barilli A. Visigalli R. Ferrari F. Frati C. Lagrasta C. (2020). Characterization of ABC transporters in EpiAirway^TM^, a cellular model of normal human bronchial epithelium. Int. J. Mol. Sci. 21 (9), 3190. 10.3390/ijms21093190 32366035 PMC7247561

[B77] Sadekar N. Behrsing H. P. Hansen T. Patel V. Paulo H. Rae A. (2025). A proof-of-concept for safety evaluation of inhalation exposure to known respiratory irritants using *in vitro* and *in silico* methods. Toxics 13 (1), 35. 10.3390/toxics13010035 39853033 PMC11769436

[B78] Sauer U. G. Vogel S. Hess A. Kolle S. N. Ma-Hock L. van Ravenzwaay B. (2013). In vivo-in vitro comparison of acute respiratory tract toxicity using human 3D airway epithelial models and human A549 and murine 3T3 monolayer cell systems. Toxicol Vitro 27 (1), 174–190. 10.1016/j.tiv.2012.10.007 23085368

[B6] SCCS (2019). Opinion of the scientific committee on consumer safety (SCCS) – final opinion on the safety of fragrance ingredient acetylated vetiver oil (AVO) - (vetiveria zizanioides root extract acetylated) - submission III. Regul. Toxicol. Pharmacol. 107, 104389. 10.1016/j.yrtph.2019.05.014 31176744

[B79] SCCS (2024). SCCS opinion on the inhalation toxicity of the fragrance ingredient acetylated vetiver oil – AVO (CAS no 84082-84-8, EC no 282-031-1) in sprayable cosmetic products - Sub - European commission. Available online at: https://health.ec.europa.eu/publications/sccs-opinion-inhalation-toxicity-fragrance-ingredient-acetylated-vetiver-oil-avo-cas-no-84082-84-8_en (Accessed May 30, 2025).

[B80] Schimek K. Frentzel S. Luettich K. Bovard D. Rütschle I. Boden L. (2020). Human multi-organ chip co-culture of bronchial lung culture and liver spheroids for substance exposure studies. Sci. Rep. 10 (1), 7865. 10.1038/s41598-020-64219-6 32398725 PMC7217973

[B81] Sharma M. Stucki A. O. Verstraelen S. Stedeford T. J. Jacobs A. Maes F. (2023). Human cell-based *in vitro* systems to assess respiratory toxicity: a case study using silanes. Toxicol. Sci. 195, 1–41. 10.1093/toxsci/kfad074 37498623 PMC10535780

[B82] Sharma M. Huber E. Arnesdotter E. Behrsing H. P. Bettmann A. Brandwein D. (2025). Minimum information for reporting on the TEER (trans-epithelial/endothelial electrical resistance) assay (MIRTA). Archives Toxicol. 99 (1), 57–66. 10.1007/s00204-024-03879-z 39365315 PMC11742365

[B83] Shrestha J. Razavi Bazaz S. Aboulkheyr Es H. Yaghobian Azari D. Thierry B. Ebrahimi Warkiani M. (2020). Lung-on-a-chip: the future of respiratory disease models and pharmacological studies. Crit. Rev. Biotechnol. 40 (2), 213–230. 10.1080/07388551.2019.1710458 31906727

[B84] Sima M. Cervena T. Elzeinova F. Ambroz A. Beranek V. Vojtisek-Lom M. (2022). The impact of extractable organic matter from gasoline and alternative fuel emissions on bronchial cell models (BEAS-2B, MucilAir^TM^). Toxicol. Vitro 80, 105316. 10.1016/j.tiv.2022.105316 35066112

[B85] Simon S. Cantrill C. Lehr C.-M. (2025). “The role of ABC transporters in the human lung Epithelium—Insights from and limitations of current *in vitro* cell models,” *in Vitro* Models. 10.1007/s44164-025-00091-w 0123456789 PMC12957684 41788234

[B86] Song Y. Namkung W. Nielson D. W. Lee J. W. Finkbeiner W. E. Verkman A. S. (2009). Airway surface liquid depth measured in *ex vivo* fragments of pig and human trachea: dependence on na+ and Cl-channel function. Am. J. Physiology - Lung Cell. Mol. Physiology 297 (6), 1131–1140. 10.1152/ajplung.00085.2009 19820035 PMC2793186

[B87] Sørli J. B. Sengupta S. Jensen A. C. Ø. Nikiforov V. Clausen P. A. Hougaard K. S. (2022). Risk assessment of consumer spray products using *in vitro* lung surfactant function inhibition, exposure modelling and chemical analysis. Food Chem. Toxicol. 164, 112999. 10.1016/j.fct.2022.112999 35427705

[B88] Steiling W. Bascompta M. Carthew P. Catalano G. Corea N. D’Haese A. (2014). Principle considerations for the risk assessment of sprayed consumer products. Toxicol. Lett. 227 (1), 41–49. 10.1016/j.toxlet.2014.03.005 24657525

[B89] Stival A. C. S. da Silva A. C. G. Valadares M. C. (2025). Qualitative and quantitative evaluation of fetal bovine serum composition: toward ethical and best quality *in vitro* science. NAM J. 1, 100047. 10.1016/j.namjnl.2025.100047 PMC13289150 42369414

[B90] Stucki A. O. Stucki J. D. Hall S. R. R. Felder M. Mermoud Y. Schmid R. A. (2015). A lung-on-a-chip array with an integrated bio-inspired respiration mechanism. Lab. Chip 15 (5), 1302–1310. 10.1039/C4LC01252F 25521475

[B91] Stucki A. O. Sauer U. G. Allen D. G. Kleinstreuer N. C. Perron M. M. Yozzo K. L. (2024). Differences in the anatomy and physiology of the human and rat respiratory tracts and impact on toxicological assessments. Regul. Toxicol. Pharmacol. 150, 105648. 10.1016/j.yrtph.2024.105648 38772524 PMC11198871

[B92] Tarran R. Grubb B. R. Gatzy J. T. Davis C. W. Boucher R. C. (2001). The relative roles of passive surface forces and active ion transport in the modulation of airway surface liquid volume and composition. J. General Physiology 118 (2), 223–236. 10.1085/jgp.118.2.223 11479349 PMC2233832

[B93] Tratnjek L. Sibinovska N. Kristan K. Kreft M. E. (2021). *In vitro* ciliotoxicity and cytotoxicity testing of repeated chronic exposure to topical nasal formulations for safety studies. Pharmaceutics 13 (11), 1750. 10.3390/PHARMACEUTICS13111750 34834166 PMC8618987

[B94] US EPA (2021). Chlorothalonil: revised human health draft risk assessment for registration review. Available online at: https://www.regulations.gov/document/EPA-HQ-OPP-2011-0840-0080 (Accessed June 25, 2025).

[B95] van der Zalm A. J. Barroso J. Browne P. Casey W. Gordon J. Henry T. R. (2022). A framework for establishing scientific confidence in new approach methodologies. Archives Toxicol. 96 (11), 2865–2879. 10.1007/s00204-022-03365-4 35987941 PMC9525335

[B96] Viegas J. Cardoso E. M. Bonneau L. Esteves A. F. Ferreira C. L. Alves G. (2024). A novel bionebulizer approach to study the effects of natural mineral water on a 3D *in vitro* nasal model from allergic rhinitis patients. Biomedicines 12 (2), 408. 10.3390/biomedicines12020408 38398010 PMC10886703

[B97] Wallace J. Jackson G. R. Kaluzhny Y. Ayehunie S. Lansley A. B. Roper C. (2023). Evaluation of *in vitro* rat and human airway epithelial models for acute inhalation toxicity testing. Toxicol. Sci. 194 (2), 178–190. 10.1093/toxsci/kfad058 37280087

[B98] Wallace J. McElroy M. C. Klausner M. Corley R. Ayehunie S. (2025). Two- and three-dimensional culture systems: respiratory *in vitro* tissue models for chemical screening and risk-based decision making. Pharmaceuticals 18 (1), 113. 10.3390/PH18010113 39861174 PMC11768377

[B99] Welch J. Wallace J. Lansley A. B. Roper C. (2021). Evaluation of the toxicity of sodium dodecyl sulphate (SDS) in the MucilAir^TM^ human airway model *in vitro* . Regul. Toxicol. Pharmacol. 125, 105022. 10.1016/J.YRTPH.2021.105022 34333067

[B100] Zamprogno P. Wüthrich S. Achenbach S. Thoma G. Stucki J. D. Hobi N. (2021). Second-generation lung-on-a-chip with an array of stretchable alveoli made with a biological membrane. Commun. Biol. 4 (1), 168. 10.1038/s42003-021-01695-0 33547387 PMC7864995

